# Interictal osmophobia is associated with longer migraine disease duration

**DOI:** 10.1186/s10194-022-01451-7

**Published:** 2022-07-15

**Authors:** Gudrun Gossrau, Marie Frost, Anna Klimova, Thea Koch, Rainer Sabatowski, Coralie Mignot, Antje Haehner

**Affiliations:** 1 University Pain Center Dresden, University Hospital and Faculty of Medicine Carl Gustav Carus, TU Dresden, Fetscherstr. 74, 01307 Dresden, Germany; 2 NCT Partner Site Dresden, Institute for Medical Informatics and Biometrics, Faculty of Medicine Carl Gustav Carus, TU Dresden, Dresden, Germany; 3 Department of Anesthesiology and Intensive Care Medicine, University Hospital and Faculty of Medicine Carl Gustav Carus, TU Dresden, Dresden, Germany; 4 Department of Otorhinolaryngology, Smell & Taste Clinic, TU Dresden, Dresden, Germany

**Keywords:** Osmophobia, Migraine, Migraine disorders, Migraine headache, Sensitization, MIDAS

## Abstract

**Background:**

Sensitization to sensory stimuli is an essential feature of migraine attacks. The relationship between the clinical course of migraine and increased sensitivity to olfactory stimuli has been little studied so far.

**Methods:**

We analyzed the frequency and quality of osmophobia depending on the phase of migraine in patients with episodic and chronic migraine treated in an tertiary headache center with regard to gender, age, medical history and migraine disability assessment score (MIDAS). Standardized diagnostic questions were used for the assessment of osmophobia.

**Results:**

In our cross-sectional investigation (*n* = 113), 38.1% of the patients showed an increased preictal hypersensitivity to odors, whereas 61.9% described ictal and 31.9% interictal hypersensitivity to odors, odor-triggered migraine was described in 30.1%. Median migraine disease duration has been statistically significantly longer in patients who suffered from interictal hypersensitivity to odors (28.5 years vs. 20 years; *p* = 0.012). There was a significant correlation between interictal hypersensitivity and higher age (54.50 vs. 45; *p* = 0.015). Patients with higher migraine disability in MIDAS experienced more frequently preictal and interictal olfactory sensitization and odor triggered migraine attacks.

**Conclusions:**

In patients with longer migraine disease duration and higher migraine-related impairment, osmophobia was more frequently observed. These results might support the hypothesis of increasing sensitization with increasing burden of migraine.

## Background

Migraine results from episodic functional changes of cerebral networks [1]. This is accompanied by altered perception of a variety of sensory stimuli [2] and becomes clinically evident with photophobia, phonophobia and osmophobia. Odor triggered migraine attacks have been suggested as a potentially useful diagnostic criterion for migraine [3].

Several studies indicated that migraineurs present with deviations in sensory processing not only ictally but also interictally. In detail, pronounced neuronal sensitization has been shown in ictal migraineurs using visual evoked potentials [4]. Sensitization for auditive stimuli was demonstrated ictally and interictally in patients with migraine by quantitative measurement of sound-induced discomfort and pain [5]. In terms of olfaction, migraine patients with or without osmophobia exhibit higher olfactory thresholds than healthy controls [6] which actually indicates an impaired olfactory sensitivity in migraine. Osmophobia is defined as the aversion or hypersensitivity to odors. Previous data suggest that those patients who regarded themselves as hypersensitive to odors between attacks presented with a higher frequency of attacks and higher number of odor-induced migraines [7]. Using positron emission tomography in these patients, a specific role of the piriform cortex and the antero-superior temporal gyrus in odor triggered migraine has been suggested [8]. Further, odor stimulation during an fMRI experiment in acute migraine attacks induced an increased activity of the rostral pons, a structure involved in the trigemino-nociceptive pathway in migraine pathophysiology pointing towards a close link between olfaction and pain [9].

The prevalence of osmophobia in migraine has been reported to be very high whereas published data differ widely between 24.7% and 95.5% [10–12]. A study in 1170 patients with primary headaches showed, that osmophobia is common in patients with migraine and its subtypes, but was absent in patients with episodic tension type headache and cluster headache. Headache patients with osmophobia showed longer headache duration, more severe anxiety, depression allodynia and headache intensity [13]. A questionnaire-based study focusing on olfaction in migraineurs described odor-related disturbances as common symptoms and osmophobic complaints in almost all participants [11]; a recent study however, observed osmophobia in only 30% of migraineurs [14]. Additional data report that osmophobia and a marker of central sensitization, cutaneous allodynia, are more frequent in chronic than in episodic migraineurs with a significant correlation between these symptoms in patients with chronic migraine [15].

According to the international classification of headache disorders, chronic migraine (cM) is defined as at least 15 headache days per month over the preceding 3 months with migraine features present on at least 8 days per month [16]. Migraine chronification, involves a variety of risk factors, such as fequency of migraine attacks, overuse of analgesics and comorbid pain disorders. The concept of migraine stages describes a gradual transition from low to high frequent episodic migraine and cM (0–9; 10–14 and ≥ 15 headache days per month, respectively) [17].

Recent results suggest comorbidities and environmental rather than genetic factors [18] as keys of chronification in migraine patients. In line with this, depression and anxiety are more common in chronic than in episodic migraine and especially the presence of comorbid depression is associated with higher risk of developing cM [19]. Until now, investigations on the clinical course and the presence of osmophobia as indicator of olfactory sensitization in patients with migraine have been very rare. The aim of this study was to analyze the frequency and quality of osmophobia in episodic and chronic migraine patients with and without aura with respect to migraine-related disability, disease duration, concomitant diseases and co-medication.

## Methods

The study protocol was approved by the Ethics Board of the Faculty of Medicine at the TU Dresden (protocol number EK-264062020). Detailed information about the experiment was given to all participants and informed written consent was obtained. All aspects of the study were performed in accordance with the Declaration of Helsinki. The authors collected and had access to the patients data. There were no missing data for the osmophobia questions.

### Participants and clinical data

In total, 113 patients with episodic or chronic migraine with or without aura were included in this cross-sectional study (see Table 1). No statistical power calculation was conducted prior to the study. The sample size was based on the available data of individuals presenting for treatment. All patients were out-patients at the Headache Clinic of the TU Dresden Pain Center. Diagnoses were confirmed according to the International Classification of Headache Disorders III by a neurologist specialized in headache. The median age was 48 years old for women (range 19–78; mean age 46,8 ± 13,9) and 41 years old for men (range 18–66; mean age 40,9 ± 14,2). For migraine with aura the median age was 44 (range 22–68; mean age 43,5 ± 12,5) and 49 (range 18–78; mean age 48,1 ± 14,9) years old for migraine without aura.

**Table 1:** Clinical characteristics of patients with migraine

	n (%)
*Patients with Migraine:*	113 (100)
female	99 (87.6)
male	14 (12.4)
*Migraine:*	113 (100)
Migraine with Aura	50 (44.2)
Migraine without Aura	63 (55.8)
Episodic Migraine	95 (84.1)
Chronic Migraine	18 (15.9)
*Concomitant Tension type headache:*	
Sporadic	15 (13.3)
Episodic	8 (7.1)
Chronic	2 (1.8)
Concomitant disease:	
Internal disease	45 (39.8)
Orthopaedic disorders	22 (19.5)
Cancer	3 (2.7)
Concomitant Mental disorder:	
Anxiety disorder	3 (2.7)
Depression	20 (17.7)
Concomitant pain disorders:	
Chronic Cervical pain	25 (22.1)
Chronic Thoracic pain	3 (2.7)
Chronic lower back pain	22 (19.5)
Analgesics used in migraine attacks:	
Ibuprofen	42 (37.2)
Acetylsalicylic acid	13 (11.5)
Triptans	69 (61.1)
Dipyrone	10 (8.8)
Paracetamol/Acetaminophen	14 (12.4)
Dimenhydrinat	1 (0.9)
Migraine prophylactic medication:	
Beta-blockers	17 (15.0)
Topiramate	5 (4.4)
Flunarizine	3 (2.7)
Amitryptiline	5 (4.4)
Onabotulinumtoxin A	1 (0.9)
monoclonal antibodies against CGRP/CGRP-receptor	2 (1.8)
Age in years	46 ± 14 (range: 18–78)
Women	47 ± 14 (range: 19–78)
Men	41 ± 14 (range: 18–66)
Migraine with aura	44 ± 13 (range: 22–68)
Migraine without aura	48,11 ± 15 (range: 18–78)
Episodic migraine	46 ± 14 (range: 18–78)
Chronic migraine	48 ± 12 (range: 24–66)
BMI in kg/m^2^	24.4 ± 4.4 (range: 17.2–43.4)
Women	24.3 ± 4.6 (range: 17.2–43.4)
Men	24.5 ± 3.3 (range: 19.8–31.2)
Migraine with aura	25.5 ± 4.8 (range: 18.1–43.4)
Migraine without aura	23.5 ± 3.9 (range: 17.2–38.4)
Episodic migraine	24.2 ± 4.4 (range: 17.2–43.4)
Chronic migraine	24.9 ± 4.5 (range: 18.8–33.5)

Age and BMI described as Mean ± standard deviation (SD)

### Questionnaires

As part of their routine clinical assessment, patients provided information on their self-perceived preictal, ictal and interictal olfactory sensitivity and on odor-triggered migraine attacks. In detail, the patients were asked four questions for the anamnestic assessment of osmophobic behavior: “Question 1) Are you sensitive to odors before a migraine attack?”, “Question 2) Are you sensitive to odors during a migraine attack?”, “Question 3) Are you sensitive to odors on days in between migraine attacks?” and “Question 4) Can odors trigger migraine attacks in you?“. In case of a positive answer, the patients named all scents to which they are sensitive or which could be a triggering factor. The questions have been developed by AH and GG based on patients feedback about olfactory sensitivity in migraine and on clinical experience in the field of olfaction and headache medicine. Olfactory sensitivity in different phases of the migraine cycle were intended to be reported. In a face-to-face interview, the reliability of the patient information was validated, especially after explanation of osmophobia that is increased sensitivity to odors. Additional clinical data were obtained from medical history: age, gender, height and weight, disease duration, migraine-related disability in everyday life, migraine with aura, concomitant diseases and co-medication.

Migraine-related disability has been assessed using the validated migraine disability assessment score (MIDAS [20]). Patients were classified into two categories: 1-high migraine-related impairment, 2-low/moderate migraine-related impairment. The cut-off value for the MIDAS was 21 points. Patients, who exceeded the cut-off value were assigned to the group “high migraine-related impairment”.

### Statistical analyses

For the primary and preplanned analysis of patient data on the migraine factors were summarized using frequency tables for the following categorical variables: migraine impairment (low/severe), odor as migraine trigger (yes/no), migraine with aura (yes/no), chronic migraine (yes/no), odor sensitivity (yes/no). The association within each pair of these variables was analyzed using the chi-squared test of independence. The normality of continuous outcomes, namely, disease duration and number of migraine days, was tested by using the Kolmogorov-Smirnov test. If the data were normally distributed, the between-group differences were examined using a two-sample t-test, otherwise the Mann-Whitney-U test was performed. The data analysis was carried out using Statistical Package for the Social Sciences (SPSS) for Windows SPSS (Version 27.0). In hypothesis testing the significance level of α = 0.05 (two-sided) was used, and *p*-values lower than this level were considered to be statistically significant. When appropriate, a Bonferroni correction was applied.

## Results

### Frequency and quality of olfactory sensitization in migraineurs

Preictal hypersensitivity to odors was reported in 38.1% of patients with migraine. Ictal hypersensitivity to odors was reported in 61.9% of patients with migraine. Interictal hypersensitivity to odors on days without migraine was reported in 31.9% of patients with migraine. In 30.1% of patients, odors commonly induced migraine attacks (Table 2).

**Table 2:** Olfactory hypersensitivity in *n* = 113 patients with migraine

Characteristics	n (%)
preictal hypersensitivity: yes / no	43 (38,1) / 70 (61,9)
ictal hypersensitivity: yes / no	70 (61,9) / 43 (38,1)
interictal hypersensitivity: yes / no	36 (31,9) / 77 (68,1)
odor-triggered migraine attacks: yes / no	34 (30,1) / 79 (69,9)
Migraine-related impairment in daily life: high/moderate-low	45 (39,8) / 68 (60,2)

**Fig. 1:**
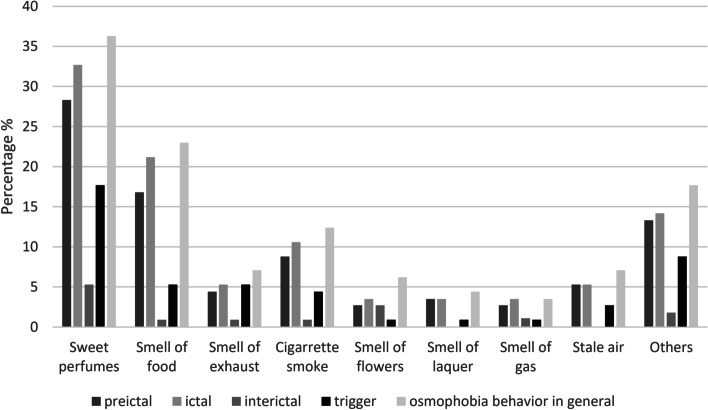
Frequencies of the main odorants involved in osmophobia. The questions asked to investigate osmophobia were: “Question 1) Are you sensitive to odors before a migraine attack?”, “Question 2) Are you sensitive to odors during a migraine attack?”, “Question 3) Are you sensitive to odors on days without migraine?” and “Question 4) Can odors trigger migraine attacks in you?“

Not all patients could name the odors regarding to the four questions of osmophobia behavior. Perfumes, food odors, and smoke were the most commonly named odors for osmophobia behavior in general (Fig. 1).

As patients may present any combination of three sensitivity types, the corresponding frequencies were summarized using a three-way contingency table, and the association was investigated using a log-linear model that includes all pairwise interactions between sensitivity types, namely, preictal*ictal, ictal*interictal, and preictal*interictal (Table 3). The interaction between preictal and ictal sensitivity was found to be very strong (OR = 10.611, 95%CI [3.355; 33.557], *p* < 0.001), and so was the interaction between ictal and interictal sensitivity (OR = 3.546, 95%CI [1.218; 10.324], *p* = 0.020). The interaction between preictal and interictal was weaker and not statistically significant (OR = 1.789, 95%CI [0.723; 4.429], *p* = 0.208). The association of odor-triggered migraine attacks with preictal sensitivity (OR = 2.896, 95%CI [1.139, 7.364], *p* = 0.026) and with interictal sensitivity (OR = 4.419, 95%CI[1.783; 10.952], *p* = 0.001) turned out to be significant, although the association with ictal sensitivity was not significant (OR = 1.737, 95%CI [0.556; 5.423], *p* = 0.342).

**Table 3:** Migraine diagnosis and olfactory sensitization in* n* = 113 patients with migraine. Migraine-related impairment in daily life: evaluation in categories: high vs. low impairment - cut-off value for MIDAS 21 points

	interictal	odour triggered	preictal	ictal	Midas category	Midas numeric	disease duration	age	BMI
interictal hypersensitivity									
odour triggered attack	OR = 4.419 [1.783;10.952] ***p*** **= 0.001**								
preictal hypersensitivity	OR = 1.789 [0.723;4.429] *p* = 0.208	OR = 2.896 [1.139;7.364] ***p*** **= 0.026**							
ictal hypersensitivity	OR = 3.546 [1.218;10.324] ***p*** **= 0.02**	OR = 1.737 [0.556;5.423] *p* = 0.342	OR = 10.611 [3.355;33.557] ***p*** **< 0.001**						
Midas category	OR = 1.814 [0.754;4.364] *p* = 0.184	OR = 1.909 [0.807;4.516] *p* = 0.141	OR = 2.653 [1.061;6.633] ***p*** **= 0.037**	OR = 0.627 [0.25;1.57] *p* = 0.318					
Midas numeric	OR = 2.007 [1.156; 3.484] ***p*** **= 0.015**	OR = 1.940 [1.133;3.320] ***p*** **= 0.017**	OR = 1.554 [0.879; 2.748] *p* = 0.132	OR = 0.914 [0.512; 1.631] *p* = 0.761					
disease duration	M = 8.036 [1.666; 14.406] ***p*** **= 0.015**	M = 2.119 [− 4.091; 8.330] *p* = 0.505	M = − 1.745 [− 8.656; 5.166] *p* = 0.622	M = 0.85 [− 6.152;7.852] *p* = 0.445	W = 1042.5 *p* = 0.435	Spearman Rho = 0.140 *p* = 0.170			
age	M = 10.7 [3.938; 17.462] ***p*** **= 0.002**	M = 1.346 [− 4.325; 7.017] *p* = 0.643	M = 0.507 [− 5.412; 6.426] *p* = 0.867	M = − 4.7 [− 11.913; − 2.513] *p* = 0.204	W = 1642 *p* = 0.513	Spearman Rho =0.180 ***p*** **= 0.056**	Spearman Rho = 0.651 ***p*** **< 0.001**		
BMI	M = − 1.512 [− 3.401;0.377] *p* = 0.120	M = 0.6777 [− 1.216;2.572] *p* = 0.479	M = 2.487 [0.491;4.483] ***p*** **= 0.016**	M = 0.588 [− 1.377;2.554] *p* = 0.559	W = 1366.5 *p* = 0.543	Spearman Rho = 0.134 *p* = 0.179	Spearman Rho = 0.141 *p* = 0.186	Spearman Rho = 0.258 ***p*** **= 0.009**	
chronic migraine diagnosis	OR = 1.226 [0.394;3.815] *p* = 0.725	OR = 0.619 [0.165;1.896] *p* = 0.431	OR = 1.292 [0.389;4.288] *p* = 0.675	OR = 0.607 [0.177; 2.079] *p* = 0.426	*χ*^2^(1) = 6.009 ***p*** **= 0.014**	W = 1338 ***p*** **< 0.001**	W = 645 *p* = 0.920	W = 964 *p* = 0.394	W = 747 *p* = 0.590

The association between odor sensitivity with each of MIDAS (high/low), migraine with aura, and odor-triggered migraine attacks was also studied using a log-linear model, which, in each case, included the pairwise interaction of the variable of interest with odor sensitivity, as well as the preictal*ictal and interictal*ictal interactions. The interaction of MIDAS with preictal sensitivity was found statistically significant (OR = 2.653, 95%CI[1.061; 6.633], *p* = 0.037), while its interaction with ictal sensitivity (OR = 0.627, 95%CI [0.250; 1.570], *p* = 0.318) and with interictal sensitivity (OR = 1.814, 95%CI [0.754; 4.364], *p* = 0.184) were not significant. Similarly, no association between olfactory sensitivity types and migraine with aura was detected (Table 4).

**Table 4:** Migraine diagnosis and olfactory sensitization in *n* = 113 patients with migraine

	Osmophobia during migraine cycle
Migraine (with aura/without aura)	preictal hypersensitivity Migraine with aura: 24 (21.2%) Migraine without aura: 19 (16.8%) ictal hypersensitivity Migraine with aura: 36 (31.9%) Migraine without aura: 34 (30.1%) interictal hypersensitivity Migraine with aura: 16 (14.2%) Migraine without aura: 20 (17.7%) odor-triggered migraine attacks Migraine with aura: 18 (15.9%) Migraine without aura: 16 (14.2%)
Migraine (Chronic migraine/episodic migraine)	preictal hypersensitivity Chronic migraine: 7 (6.2%) Episodic migraine: 36 (31.9%) ictal hypersensitivity Chronic migraine: 10 (8.8%) Episodic migraine: 60 (53.1%) interictal hypersensitivity Chronic migraine: 6 (5.3%) Episodic migraine: 30 (26.5%) odor triggered migraine attacks Chronic migraine: 4 (3.5%) Episodic migraine: 30 (26.5%)
Disease duration (Median, years)	preictal hypersensitivity *x* ~(preictal hypersensitivity) = 20.0 years *x* ~(no preictal hypersensitivity) = 21.0 years ictal hypersensitivity *x* ~(ictal hypersensitivity) = 20.0 years *x* ~(no ictal hypersensitivity) = 21.0 years interictal hypersensitivity *x* ~ (interictal hypersensitivity) = 28.5 years *x* ~ (no interictal hypersensitivity) = 20 years odor-triggered migraine attacks *x* ~(odor-triggered migraine attacks) = 20.0 years *x* ~(no odor-triggered migraine attacks) = 21.0 years
Migraine disability (Median, Midas-score)	preictal hypersensitivity *x* ~(preictal hypersensitivity) = 32.00 *x* ~(no preictal hypersensitivity) = 21.50 ictal hypersensitivity *x* ~(ictal hypersensitivity) = 31.00 *x* ~(no ictal hypersensitivity) = 23.00 interictal hypersensitivity *x* ~(interictal hypersensitivity) = 37.00 *x* ~(no interictal hypersensitivity) = 23.00 odor-triggered migraine attacks *x* ~(odor-triggered migraine attacks) = 36.00 *x* ~(no odor-triggered migraine attacks) = 23.00

### Migraine duration and olfactory sensitization

The relationship between odor sensitivity types and disease duration was explored using the analysis of variance (ANOVA). No statistically significant difference in disease duration was found regarding preictal sensitivity (t(94) = − 0.945; *p* = 0.622) or ictal hypersensitivity to odors (t(94) = 0.238; *p* = 0.813). However, patients with interictal sensitivity are characterized by on average 8 years longer disease duration than those without (t(94) = 2.473, *p* = 0.015). No significant difference in disease duration between patients with and without odor-triggered migraine attacks were found (t(96) = 0,669; *p* = 0,505), see Tables 3 and 4.

The age effect on olfactory sensitization was also studied using ANOVA. A significant age difference was found only with respect to the interictal sensitivity. Patients who showed interictal osmophobia were on average 10 years older than those who did not (t(108) = 3.103, *p* = 0.002). For preictal and ictal sensitivity, as well for odor-triggered migraine attacks, there was no significant age difference.

BMI data were available for 102 out 113 study participants. Only the difference between patients with and without preictal sensitivity was found significant (t(98) = 2.442, *p* = 0.016).

No significant association between osmophobia behavior and concomitant pain diseases, diagnosed affective disorders or analgesic intake have been found (for details see Tables 1 and 4).

### Migraine-related disability and osmophobia

A high migraine-related disability, as measured by the MIDAS-Score, was presented in 60.2% of 113 patients, for whom the MIDAS data were available. Patients with chronic migraine (cM) and episodic migraine (eM) exhibited different MIDAS (MIDAS-Score: cM = 92.50 vs. eM = 23.00). MIDAS score in patients with cM was statistically significantly higher than in patients with eM (U = 376.500; z = − 3.755; *p* = 0.000).

According to their numerical values of MIDAS, the study participants were categorized as having a high or low migraine-related disability. The association between disability status and odor sensitivity was analyzed using a log-linear model. Based on the fit results, preictal hypersensitivity occurred more likely in patients with high migraine-related disability than in those with lower disability (OR = 2.653 [1.061; 6.633], *p* = 0.037), but no significant association between this disability and ictal or interictal sensitivity was found (OR = 0.627 [0.250; 1.570], *p* = 0.318, and OR = 1.814 [0.754; 4.364], *p* = 0.184, respectively). Finally, there was no significant difference regarding to migraine-related disability between patients with and without odor-triggered migraine attacks (OR = 1.898 [0.753; 5.069], *p* = 0.203).

**Fig. 2:**
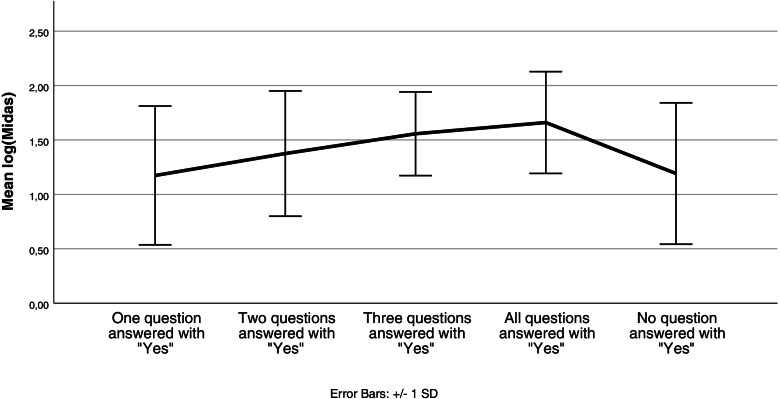
Mean migraine disability score and osmophobia behavior***.*** The questions asked to investigate osmophobia were: “Question 1) Are you sensitive to odors before a migraine attack?”, “Question 2) Are you sensitive to odors during a migraine attack?”, “Question 3) Are you sensitive to odors on days without migraine?” and “Question 4) Can odors trigger migraine attacks in you?”

The difference in osmophobia behavior was also investigated using the numerical MIDAS sumscores (Fig. 2). High MIDAS-scores have been obtained by patients who declared preictal olfactory sensitization (Table 4, MIDAS-Score: preictal hypersensitivity = 32.00 vs. no preictal hypersensitivity = 21.50; (U = 1160.500; z = − 2.038; *p* = 0.042)), ictal olfactory sensitization (MIDAS-Score: ictal hypersensitivity = 31.00 vs. no ictal hypersensitivity = 23.00; (U = 1269.500; z = − 1.393; *p* = 0.164)), interictal hypersensitivity to odors (MIDAS-Score: interictal hypersensitivity = 37.00 vs. no interictal hypersensitivity = 23.00; (U = 915.500; z = − 2.900; *p* = 0.004)) and odor triggered migraine attacks (MIDAS-Score: odor triggered migraine attacks = 36.00 vs. no odor triggered migraine attacks = 23.00; (U = 978.000; z = − 2.285; *p* = 0.022)). To account for multiple sensitization types per patient, the (log10) MIDAS-scores were studied using the analysis of variance. With this approach, no significant differences were identified, except for a borderline significant effect of the interictal sensititvity (t(108) = 2.016, *p* = 0.0462). Significantly higher Midas-scores have been obtained by patients who declared preictal, interictal olfactory sensitization and odor triggered migraine attacks (Fig. 2).

## Discussion

The results of our study provide further evidence for the close relationship between migraine and olfaction.

We found that more than 50% of patients with migraine presented with preictal/ictal osmophobia which is in line with recently published data of a prospective investigation [21]. Odor-triggered migraine attacks were less commonly described and associated however, with osmophobia. In patients with migraine, it was shown in several publications, that subjective hypersensitivity to odors is more common not only during migraine attacks but also interictally. Here, parallels were drawn to olfactory hypersensitivity because self-assessment in patients with migraine associated with findings in a chemical odor intolerance test [8]. Further, an interview-based study in 1750 migraine patients reported in 43.7% of them perfume or a specific odor as a trigger of migraine attacks [10]. In our study, the percentage of patients with odor-induced attacks was lower (25%), which could be due to the use of questionnaires rather than interviews. This might have resulted in a lower reported number when patients were not reflecting odors as trigger or vice versa, a non-structured interview with suggestive questions could result in an artificially high prevalence.

A statistically significant association between migraine-related disability and osmophobia has been found. However, osmophobia has not been significantly more common in patients with cM, even though cM is accompanied by higher migraine-related disability. This discrepancy might be due to the small number of cM patients. Considering the disease severity, our results are supported by Kelman et al. who indicated in a study with > 1700 migraineurs that odor/perfume triggered migraine attacks were more common in chronic migraine patients [10]. Published data show an association between higher attack frequency and a higher number of odor-induced migraines in patients who assessed themselves as olfactory hypersensitive [8]. This is in line with our finding, which showed that migraine-related disability caused by frequent migraine attacks and osmophobia are features that are more likely associated.

Our study depicts a relation between disease duration and olfactory sensitization. This might support the hypothesis, that longer disease duration increases sensory sensitization and therefore, the risk of chronification. In line with this, a study in 1170 patients with primary headaches reported, that headache patients with osmophobia in at least 20% of the headache episodes showed longer headache duration and more often allodynia and higher headache intensities [13]. Another study however, did not find an association between disease duration and interictal olfactory hypersensitivity [8]. Data on subjective sensory hypersensitivity in 187 patients with migraine showed not only a common overlap of subjective sensory hypersensitivities to light, noise and smell but also a clear relation between sensory hypersensitivities and migraine disability [22].

Pre/ictal osmophobia has so far not been investigated in this context. Based on these results, one could put forward the hypothesis that smell loss (eg. postinfectious or idiopathic) might induce an olfactory desensitization in migraine patients. However, currently there is no evidence that olfactory impairment is accompanied by a decrease in osmophobia or odor-triggered attacks. Recently, we conducted a brief telephone interview in 16 patients with smell loss and a history of migraine. Seven out of them reported osmophobia and all but one additional odor-triggered attacks without any changes in intensity, frequency, and character after the onset of smell loss. This can be attributed to a residual smell function (only one patients was a functional anosmic) or might on the other hand point towards a severe chronification of this patient group or indicate a missing relation to the objective olfactory performance [6].

The most common migraine triggering odors in our study were sweet perfumes, food and cigarette odors. This is in line with recent prospective study data, which report perfumes followed by cigarette and/or cigar smoke and by food as most common olfactory migraine triggers [21] and earlier studies referring to perfumes, smoke and cleaning products [23–26]. These odors are found very frequently in the environment and are characterized by strong hedonic valences.

Imaging studies suggested activation of common neuronal structures upon olfactory and nociceptive stimulation. In addition, activation of olfactory neurons has impact on the default mode network of the brain [27]. Furthermore, rostral pons has been found to be activated by olfactory stimuli and is involved in migraine progression [9].

In a rat model of migraine, increased connectivity of the insular cortex and the pons, the midbrain, the thalamus, the visual and sensory cortices has been demonstrated, suggesting that chronification of migraine may be related to higher brain centers and limbic cortices [28]. Functional imaging in chronic migraine patients reported an extensive dysfunction of the pain inhibitory network and increased sensitization of central pain pathways [29, 30].

Brain areas as thalamus, hypothalamus, somatosensory and anterior cingulate cortex are thought to play a key role in migraine chronification and pain sensitization [31]. The disequilibrium between excitatory and inhibitory signals in the pain relevant brain structures yields in sensory symptoms as mechanical hyperalgesia and cutaneous allodynia [32, 33]. This is reflected by increased mechanical sensitivity in all phases of the migraine cycle compared to healthy people [34, 35].

Based on this, a growing sensory susceptibility in migraine patients may reflect the process of central nervous sensitization and chronification and increased disease load in migraine patients. Our cross-sectional study included outpatients with migraine from our tertiary center who presented for therapy. Consequently, the results are likely applicable to patients in specialized headache centers. In general neurological practice, there are fewer complex migraine patients and according to our data fewer symptoms of osmophobia are expected.

However, our study has several limitations that should be noted. First of all, the data rely on patient’s communication. A memory bias due to results obtained solely by a self-reported questionnaire cannot be excluded. In addition, maybe less pronounced associations between osmophobia and clinical parameters of migraine cannot be expressed statistically with the here presented number of patients.

## Conclusions

The here presented clinical data describe higher olfactory sensitization associated with higher burden of disease in migraine patients and support the data of sensory sensitization during the progression of migraine on a clinical level. Further studies in anosmic patients with migraine are warranted.

Based on this data, investigation of desensitizing effects of olfactory training in migraine patients will be of interest.

## Data Availability

The datasets used and/or analyzed during the current study are available from the corresponding author on reasonable request.

## Abbreviations

MIDAS: Migraine disability assessment score
fMRI: Functional Magnetic Resonance Imaging
HIT-6: Headache Impact Test
SPSS: Statistical Package for the Social Sciences
BMI: Body Mass Index
cM: Chronic Migraine
eM: Episodic Migraine
